# Severe COVID-19 virus reactivation following treatment for B cell acute lymphoblastic leukemia

**DOI:** 10.1186/s13045-020-00968-1

**Published:** 2020-10-02

**Authors:** Guido Lancman, John Mascarenhas, Michal Bar-Natan

**Affiliations:** Tisch Cancer Institute, Icahn School of Medicine At Mount Sinai, 1 Gustave Levy Place, New York, NY 10029 USA

**Keywords:** COVID-19, SARS-CoV-2, Reactivation, Rituximab, Cytarabine

## Abstract

SARS-CoV-2 has infected millions of people worldwide, but little is known at this time about second infections or reactivation. Here, we report a case of a 55-year-old female undergoing treatment for CD20+ B cell acute lymphoblastic leukemia who experienced a viral reactivation after receiving rituximab, cytarabine, and dasatinib. She was initially hospitalized with COVID-19 in April and developed a high antibody titer with two negative nasal polymerase chain reaction (PCR) swabs for SARS-CoV-2 on discharge. After recovery, she resumed treatment in June for her leukemia, which included rituximab, cytarabine, and dasatinib. She promptly lost her COVID-19 antibodies, and her nasal PCR turned positive in June. She developed a severe COVID-19 pneumonia with lymphopenia, high inflammatory markers, and characteristic bilateral ground-glass opacities on chest CT, requiring high-flow nasal cannula and transfer to the intensive care unit. She received steroids, anticoagulation, and convalescent plasma, and within 48 h she was off oxygen. She was discharged home in stable condition several days later. Given the short time frame from leukemia treatment to PCR positivity and the low case rate in mid-June in New York City, reinfection appears to have been unlikely and SARS-CoV-2 reactivation is a possible explanation. This case illustrates the risks of treating recently recovered COVID-19 patients with immunosuppressive therapy, particularly lymphocyte- and antibody-depleting therapy, and raises new questions about the potential of SARS-CoV-2 reactivation.

## To the editor

SARS-CoV-2 has currently infected over 10 million people worldwide with over 500,000 deaths. However, little is known about reactivation of SARS-CoV-2. Although positive polymerase chain reaction (PCR) for SARS-CoV-2 following two negative PCR tests has been reported in up to 14–21% of patients [1, 2], these new positive tests occurred within 30 days of the last negative test and were thought to represent prior false negative PCR results and the consequence of prolonged viral shedding. There are sporadic reports to date of clinical COVID-19 virus reactivation. Ye et al. reported 5 patients with clinical reactivation presenting mostly with fatigue and fever, but none of them developed severe COVID-19 pneumonia or died [3]. Ravioli et al. reported two elderly patients who developed COVID-19, recovered and tested negative by PCR, and then developed a new COVID-19 pneumonia, with one patient dying and the other remaining hospitalized at the time of the report [4].

Here, we report a case of severe COVID-19 virus reactivation following chemotherapy, including rituximab, cytarabine, and dasatinib for B cell acute lymphoblastic leukemia (B-ALL). A 55-year-old female with diabetes mellitus, coronary artery disease, and asthma was diagnosed with Philadelphia chromosome-positive, CD20-positive B-ALL in November of 2019. She underwent induction and consolidation per the EWALL regimen [5], with the addition of rituximab given CD20 positivity. She was in a complete remission from her leukemia when she presented to the emergency department on April 8 with fever and abdominal pain and was diagnosed with COVID-19 by nasopharyngeal PCR. As she had mild symptoms, she was discharged home; however, she was readmitted on April 20 due to persistent fevers, dry cough, abdominal pain, nausea, and vomiting. She had high inflammatory markers and bilateral ground-glass opacities on CT of the chest (Table 1). She received hydroxychloroquine and azithromycin per institution guidelines at that time with no improvement, then received remdesivir with clinical improvement, and was discharged home after 18 days with resolution of these symptoms. Upon discharge, a negative nasopharyngeal PCR for SARS-CoV-2 was documented (May 7) which was repeated 4 days later (May 11) and again confirmed negative. On May 14 she was tested for COVID-19-specific antibodies and demonstrated a high titer at 1:960.

**Table 1:** Timeline of events from initial COVID-19 diagnosis to second COVID-19 episode

Date	4/8/20	4/25/20	5/7/20	5/11/20	5/14/20	6/8/20	6/18/20	6/25/20	6/28/20
SARS-CoV-2 PCR	Positive	Positive	Negative	Negative			Positive		Positive
COVID-19 antibodies		Positive (1:80)			Positive (1:960)			Negative	
COVID-19 symptoms		Fever, cough, abdominal pain, nausea, vomiting No oxygen requirement							Fever, acute respiratory distress requiring high-flow nasal cannula
White blood cell count (× 10^3^/uL)	3.5	11.7	5.0			10.6	3.5	0.3	4.6
Absolute lymphocyte count (× 10^3^/uL)	1.9	1.4	1.6			2.6	0.2	0.1	0.1
C-reactive protein (mg/L)		168.1	9.2						314.3
Erythrocyte sedimentation rate (mm/h)									> 145
Ferritin (ng/mL)		4569	3662						> 33,511
Interleukin 6 (pg/mL)		93.4							139.0
Fibrinogen (mg/dL)		603	567						710
D-dimer (μg/mL FEU)		0.83	0.39						3.54
CT chest findings		Moderate, bilateral, scattered ground-glass opacities							Extensive, diffuse ground-glass opacities including previously unaffected sites
Leukemia treatment						Rituximab Cytarabine Dasatinib			

As she was thought to have recovered from COVID-19, she resumed consolidation therapy for B-ALL on June 8, receiving rituximab, cytarabine, and dasatinib. On June 18, she was admitted to the hospital due to fevers up to 40.3 °C (104.5 °F), sore throat, abdominal pain, bloody diarrhea, and neutropenia. SARS-CoV-2 nasopharyngeal PCR was positive; however, this was thought to be a reflection of residual viral shedding. She had a CT that showed typhlitis and marked improvement of previous lung infiltrates. She was also diagnosed with Clostridium difficile colitis at that time. Her white blood cell count reached a nadir of 0.1 × 10^3^/uL from chemotherapy on June 24. By June 27 she continued to have high fevers and developed a new cough, followed by respiratory decompensation requiring high-flow nasal cannula and transfer to the intensive care unit. Her chest CT showed extensive, diffuse ground-glass opacities at different sites from the initial COVID-19 pneumonia. Inflammatory markers were again extremely elevated (Table 1). COVID-19 antibody testing showed complete lack of COVID-19 antibodies, despite prior titer of 1:960. PCR testing remained positive on June 28. She received 2 units of convalescent plasma and dexamethasone and was proned with rapid improvement in oxygen requirements. She was weaned off high-flow nasal cannula within 48 h and within a few days was discharged home in stable condition.

The immune response toward SARS-CoV-2 comprises both the cellular and humoral arms. One recent study surveyed immune responses in 36 people recovering from COVID-19 and found T cells that recognize SARS-CoV-2 in all of them [6]. A meta-analysis of 24 studies (3,099 patients) confirmed that lymphopenia is a prevalent finding and predicts disease severity [7]. B cells capable of producing neutralizing antibodies that recognize SARS-CoV-2 were reported in people who had recovered from COVID-19, and convalescent plasma therapy produces improvements in patients' clinical symptoms and radiological and biochemical parameters [8].

Many aspects of COVID-19 remain under investigation, including possible mechanisms of reactivation. In this case, the patient had high levels of COVID-19 antibodies until treatment with rituximab, an anti-CD20 antibody that is known to deplete B cells and is a known risk factor for other viral reactivations. She also received cytarabine which caused her white blood cell count to decline to 0.1 × 10^3^/uL immediately preceding the second pneumonia. Given the short time frame from rituximab and cytarabine administration to PCR positivity and the low case rate in mid-June in New York City, reinfection appears to have been unlikely. Although there are no published data on PCR sensitivity, it is estimated to be anywhere from 70–95%. This would allow the possibility that the patient had two false negative nasopharyngeal PCR results, although this would not account for the loss of antibodies. It is also possible that the patient never fully cleared the virus from the lower respiratory tract as sputum samples have been shown to be positive longer than upper respiratory samples [9]. A different sanctuary site could be a possibility, but this has not been identified yet for SARS-CoV-2. This case illustrates the risks of treating recovered COVID-19 patients with immunosuppressive therapy, particularly lymphocyte- and antibody-depleting therapy, and raises new questions about the potential of SARS-CoV-2 reactivation.

## Data Availability

Not applicable.

## Abbreviations

B-ALL: B cell acute lymphoblastic leukemia
PCR: Polymerase chain reaction
